# The time-profile of cell growth in fission yeast: model selection criteria favoring bilinear models over exponential ones

**DOI:** 10.1186/1742-4682-3-16

**Published:** 2006-03-27

**Authors:** Peter Buchwald, Akos Sveiczer

**Affiliations:** 1 IVAX Research, Inc., 4400 Biscayne Blvd., Miami, FL 33137, USA; 2 Department of Agricultural Chemical Technology, Budapest University of Technology and Economics, 1111 Budapest, Szt. Gellért tér 4., Hungary

## Abstract

**Background:**

There is considerable controversy concerning the exact growth profile of size parameters during the cell cycle. Linear, exponential and bilinear models are commonly considered, and the same model may not apply for all species. Selection of the most adequate model to describe a given data-set requires the use of quantitative model selection criteria, such as the partial (sequential) *F*-test, the Akaike information criterion and the Schwarz Bayesian information criterion, which are suitable for comparing differently parameterized models in terms of the quality and robustness of the fit but have not yet been used in cell growth-profile studies.

**Results:**

Length increase data from representative individual fission yeast (*Schizosaccharomyces pombe*) cells measured on time-lapse films have been reanalyzed using these model selection criteria. To fit the data, an extended version of a recently introduced linearized biexponential (LinBiExp) model was developed, which makes possible a smooth, continuously differentiable transition between two linear segments and, hence, allows fully parametrized bilinear fittings. Despite relatively small differences, essentially all the quantitative selection criteria considered here indicated that the bilinear model was somewhat more adequate than the exponential model for fitting these fission yeast data.

**Conclusion:**

A general quantitative framework was introduced to judge the adequacy of bilinear versus exponential models in the description of growth time-profiles. For single cell growth, because of the relatively limited data-range, the statistical evidence is not strong enough to favor one model clearly over the other and to settle the bilinear versus exponential dispute. Nevertheless, for the present individual cell growth data for fission yeast, the bilinear model seems more adequate according to all metrics, especially in the case of *wee1*Δ cells.

## Background

During the division cycle of individual growing cells, most size-related parameters such as length (*L*), volume (*V*), surface area, dry mass and others show a continuous increase, but there is considerable controversy concerning the exact time-profile of these increases. To describe the growth period, commonly considered possibilities include linear, exponential and bilinear models, and various bodies of experimental evidence and theoretical considerations have been proposed to support one or the other [1]. The same model may not apply for all species, and because of the uncertainties in the experimental data and of the relatively small differences in predictions owing to the relatively limited data-range (approximate doubling of size during a cell cycle), it is difficult to identify the most adequate model unequivocally. Exponential models such as *V *= *αe^βt^*, which are easy to rationalize (the rate of growth is proportional to the existing size: d*V*/d*t *= *βV*) and convenient to parameterize (*α*, *β*) and implement, are often employed. However, a number of cases seem to support a bilinear-type growth pattern with growth occurring along two (or perhaps more) essentially linear segments, corresponding to constant rates, separated by a transitional period around a rate-change point (RCP) during which the rate of length-growth increases [2-9]. The difference between the two models is most evident in the time profiles of the speed (rate) of growth increases (d*L*/d*t*): that of the bilinear model contains two constant segments connected by a transition period (a characteristic sigmoid step-up function), whereas that of the exponential model shows a continuous, accelerating increase.

Whereas an exponential increase could be related to a steady growth of ribosome numbers, a bilinear pattern might be caused by effects of the cell cycle itself causing a relatively sudden rate-increase at an RCP (or more than one RCP). These effects have not yet been fully characterized. However, two different possibilities have been raised [10], one being passage through a cell-cycle stage (a so-called checkpoint) and the other being a doubling of structural genes, i.e., a "gene dosage" effect at DNA replication (S phase). A bilinear model seemed most adequate to describe the increase of cell length in fission yeast (*Schizosaccharomyces pombe*) as determined from detailed analyses of time-lapse films of single cells (wild-type, WT, and various mutants) [6,8,9]. In this cylindrical cell species, diameter does not change during the cycle; therefore, cell length is proportional to volume. The adequacy of the bilinear model has been questioned [11,12] by invoking Occam's razor, an often-used principle attributed to William of Occam (c. 1280–1349) that favors the most parsimonious model (originally *Pluralitas non est ponenda sine necessitate*, i.e., plurality should not be posited without necessity, but most often expressed as *Entia non sunt multiplicanda praeter necessitatem*, i.e., entities are not to be multiplied without necessity [13]). Accordingly, the exponential model was suggested as more adequate because it relies on fewer parameters and provides only a very slight worsening in the quality-of-fit as judged on the basis of the correlation coefficient (*r*^2^) [11]. However, when differently parameterized models are fitted to the same data, *r*^2 ^alone is not a sufficient criterion for judging adequacy, and a number of quantitative indicators (model selection criteria) such as the partial (sequential) *F*-test, the Akaike information criterion (AIC) [14,15] and the Schwarz Bayesian information criterion (SBIC) [16] can be used to decide whether or not the improvement in fitting justifies the increased number of parameters employed (i.e., whether there is enough "necessity" for "entities to be multiplied") [17-21]. Related details are briefly discussed in the Methods section.

Here, a reanalysis of the fission yeast cell growth data is presented on the basis of these more rigorous, quantitative criteria, and a general quantitative framework is introduced to judge the adequacy of bilinear versus exponential models for describing the time-profiles of arbitrary growth processes. This was also made possible by extending a recently-introduced linearized biexponential model (LinBiExp) [21] to allow fitting of general bilinear-type data with a single, unified model. Originally, LinBiExp was introduced to describe quantitative structure-activity relationship (QSAR) data such as toxicities, antimicrobial activities and receptor-binding affinities that have a maximum or a minimum, but are essentially linear sufficiently far away from the zone of the turning point (the zone of the extreme value) [21,22]. However, by extending its parameter-range, LinBiExp can easily be generalized to describe not only data that show a maximum or a minimum, but also data that show only a rate-change between two essentially linear portions, such as those presented here and related to cell growth. Because LinBiExp makes possible a smooth, continuously differentiable and fully parameterizable transition between two linear segments, it is now possible to apply a unified model in a single fitting instead of performing two separate individual linear regressions after visually separating the data into two linear portions. Hence, with LinBiExp, the minimization algorithm itself will determine the two slope values (*α*_1_, *α*_2_) and the position of the rate change point (*t*_RCP_) that result in the lowest sum of squared errors (SSE), and this no longer has to be done by the user relying on preconceived assumptions or mere visual inspection. This eliminates the error-prone and bias-sensitive procedure of performing two separate linear regressions after separating the data on the basis of visual information or some preconceived notion.

## Methods

### Data

Cell length growth data are for individual fission yeast (*Schizosaccharomyces pombe*) cells (Table 1), selected as representative during the analysis of a large number of cell cycles (40–80 for each strain). These single cell data were determined using time-lapse microscopic films and are from previous publications [8,12]. The length increases occurring during the 5 min observation periods were often less than the smallest quantifiable unit, as the resolution was 0.33 *μ*m for the wild-type and 0.13 *μ*m for the *wee1*Δ mutant cell, depending on the final magnification. As a consequence, the growth profiles tended to have stair-like patterns with a number of plateaus; these were short inside the cycle, but there was a long plateau at the end of the cycle. To obtain more uniform profiles, they were smoothed using the resistant smooth (*rsmooth*) procedure of Minitab 7.2 (Minitab, State College, PA, USA) using the default *4235H, twice *method, similar to the original publications. To verify consistency, smoothing has also been redone here with Sigma Plot 8.0 (SPSS Inc., Chicago, IL, USA) and with a 2D bisquare (1 – *u*^2^)^2 ^or Loess (1 – |*u*|^3^)^3 ^smoothing using the nearest neighbor bandwidth method and a sampling proportion of 0.3; these resulted in almost identical values. For example, average differences between the *rsmooth *and Loess values were only 0.008 *μ*m and 0.021 *μ*m for the *wee1*Δ and WT cell lines, respectively (Table 1). Data up to 135 min for the WT cell and 115 min for the *wee1*Δ cell were considered as part of the growth period and were used for fitting.

**Table 1:** Cell length data for the wild type (WT) and the *wee1*Δ mutant used for fitting

	Length *L *(*μ*m); WT cell			Length *L *(*μ*m); *wee1*Δ cell		
*Time *(min)	Measured*	Minitab rsmooth	SigmaPlot Loess	Measured**	Minitab rsmooth	SigmaPlot Loess
0	8.667	8.641	8.626	4.935	4.935	4.903
5	8.667	8.766	8.773	4.935	5.026	5.026
10	9.000	8.974	8.972	5.195	5.159	5.151
15	9.333	9.203	9.205	5.325	5.282	5.268
20	9.333	9.418	9.454	5.325	5.371	5.372
25	9.667	9.660	9.667	5.455	5.466	5.454
30	10.000	9.896	9.879	5.584	5.584	5.584
35	10.000	10.102	10.121	5.714	5.702	5.715
40	10.333	10.326	10.333	5.844	5.793	5.797
45	10.667	10.552	10.533	5.844	5.876	5.875
50	10.667	10.760	10.768	5.974	6.011	6.020
55	11.000	11.013	11.036	6.234	6.207	6.219
60	11.333	11.331	11.333	6.494	6.400	6.407
65	11.667	11.646	11.631	6.494	6.570	6.572
70	12.000	11.896	11.886	6.753	6.767	6.768
75	12.000	12.104	12.114	7.013	6.994	7.006
80	12.333	12.354	12.369	7.273	7.178	7.195
85	12.667	12.669	12.654	7.273	7.306	7.320
90	13.000	12.992	12.945	7.403	7.443	7.441
95	13.333	13.276	13.243	7.662	7.625	7.623
100	13.333	13.573	13.561	7.792	7.820	7.824
105	14.000	13.943	13.910	8.052	7.991	7.989
110	14.333	14.328	14.290	8.052	8.131	8.139
115	14.667	14.677	14.686	8.312	8.243	8.249
120	15.000	15.012	15.032	8.312	8.304	8.312
125	15.333	15.328	15.335	8.312	8.316	8.327
130	15.667	15.561	15.604	8.312	8.313	8.312
135	15.667	15.667	15.753	8.312	8.312	8.312
140	16.000	15.701	15.785	8.312	8.311	8.312
145	15.667	15.747	15.793	8.312	8.310	8.299
150	15.667	15.823	15.823	8.312	8.318	8.301
155	16.000	15.909	15.896	8.312	8.350	8.338
160	16.000	15.977	15.973	8.442	8.407	8.432
165	16.000	16.000	16.022			
170	16.000	16.000	15.998			

*Data from [12]. **Data from [8].

### Model for bilinear-type data: LinBiExp

Bilinear fitting was done with the LinBiExp model [21], which relies on the following functional form (written here as a function of time *t *instead of a general independent variable *x *and with all adjustable parameters denoted in Greek symbols):



Here *e *(*e *= 2.718...) denotes the base of the natural logarithm (ln *x *= log_*e *_*x*), and *α*_1_, *α*_2_, *χ*, *τ*_*c *_and *η *are adjustable parameters. This form is somewhat more complex than those of simple linear models, *f*(*t*) = *αt *+ *χ*, because it contains the logarithm of the sum of two exponentials, and it is not suitable for linear regression because it contains nonlinear parameters (*τ*_*c*_, *η*). Nevertheless, it allows a convenient extension of linear models with *α*_1 _and *α*_2 _representing the two different slopes and *τ*_*c *_essentially corresponding to the rate change point *t*_RCP_. LinBiExp as defined by eq. 1 is a very general bilinear model: the transition from one linear segment to the other does not necessarily have to be along a sharp break point between two lines; it can happen along a smooth, curved portion of adjustable width. The *η *parameter regulates the smoothness/abruptness of the transition between the two linear portions with smaller absolute values corresponding to more abrupt transitions [21]. Because QSAR data are usually on a decimal log-scale and are arranged to show a maximum, LinBiExp was implemented there in a slightly different form, ), and in most cases, *η *was considered as having a fixed value of 1/ln10 = 0.4343 [21,22]. No such considerations apply to the present extension; therefore, *η *is considered as an adjustable parameter, the only restriction being that its value has to remain sufficiently small to maintain a fast-enough transition between the two linear portions (i.e., to maintain an observably bilinear character over the investigated time-range, meaning that the rate of increase, d*L*/d*t*, remains constant for at least some time in both the beginning and the ending time-periods). Depending on the actual data, this might in some cases require an upper limit to be imposed on *η*, but no such restrictions were needed here. To be able to describe general bilinear data of arbitrary shapes and curvatures, *α*_1_, *α*_2 _and *η *must be allowed to take both positive and negative values; however, all of them are always positive for the present data. Thus, LinBiExp uses a novel functional form, the logarithm of the sum of two exponentials, to obtain a completely general bilinear functionality that can now fit not only data with a minimum or a maximum, such as those commonly seen in QSAR cases, but also data that show a rate-change, such as those seen for certain growth profiles.

The nonlinear fittings required for LinBiExp can be performed using either the Excel (Microsoft, Seattle, WA, USA) worksheet or the custom-built WinNonlin (Pharsight Corp., Mountain View, CA) model provided with the **model **[21] (or, obviously, any other implementation with any software capable of nonlinear regression). Those presented here were performed with WinNonlin 5.0, a software package developed for pharmacokinetic modeling [17], but well-suited for the present purposes. The Gauss-Newton (Levenberg and Hartley) minimization algorithm was used with the convergence criteria set to 10^-5^, the increment for partial derivatives set to 10^-3^, and the number of iterations set to 50. User-provided initial parameter estimates and bounds were employed. All fittings were done with unweighted data. Because LinBiExp uses a smooth, continuously differentiable functional form, the optimization process is relatively trouble-free; nevertheless, sufficient care is recommended to verify that a true and not just a local optimization minimum is reached (i.e., using an increased convergence criterion and starting with different initial parameter values from both sides of the final values). Multiple linear regressions and additional statistical analyses were performed in Excel.

### Model selection criteria

Because the various models discussed here use different numbers of parameters (*n*_par_), it is not sufficient to rely simply on the correlation coefficient *r *or its square *r*^2^:



which is a measure of the variance explained in the predicted variable *y *= *f*(*x*) and is expressed here as a function of the overall (total) variance, SS_*y *_= Σ_*i *_(*y*_*i *_- *y*_*mean*_)^2 ^and of the sum of squared errors (residual variance), SSE = Σ_*i *_(*y*_*i *_- *y*_*i*,*pred*_)^2^; it is likely to increase with an increasing number of parameters. Further discrimination between rival models (model selection criteria) is needed. Improvement (decrease) in the residual standard deviation (*s*) is a first possibility, as it accounts at least in part for the change in the degrees of freedom, *df *= *n*_obs _- *n*_par_:

*s *= (SSE/*df*)^1/2 ^    (3)

More accurate indicators (model selection criteria) include, for example, the partial (sequential) *F*-tests, Mallows's *C*_p_, the Akaike information criterion (AIC), the Schwarz Bayesian information criterion (SBIC), the minimum description length (MDL), cross validation (CV, including prediction sum of squares PRESS statistics), and Bayesian model selection [17-20]. The *F*-statistics, by using the *p*-value of the corresponding *F *probability distribution, verifies whether the reduction in SSE is statistically significant as the corresponding degrees of freedom (*df*) decrease:



The Akaike information criterion (AIC) [14,15] and the Schwarz Bayesian information criterion (SBIC) [16] were originally defined on the basis of the maximized likelihood of the model with *n*_par _parameters ():

AIC = -2ln  + 2*n*_par _    (5)

SBIC = -2ln  + *n*_par _ln(*n*_obs_)     (6)

AIC and SBIC have been used here as implemented in WinNonlin [17] (resulting from the assumption of normally distributed errors):

AIC = *n*_obs _ln(SSE) + 2*n*_par _    (7)

SBIC = *n*_obs _ln(SSE) + *n*_par _ln(*n*_obs_)     (8)

They both attempt to quantify the information content of a given set of parameter estimates by relating SSE to the number of parameters required to obtain the fit. The model associated with smaller values of AIC and SBIC is more appropriate, and, as shown by their definitions, SBIC is a more restrictive criterion on increasing *n*_par_. Sometimes, they are used in terms of ln(SSE/*n*_obs_), but for a given data-set with minimization of AIC and/or SBIC as the goal, this makes no difference. AIC is similar to Mallows's *C*_p _[23]:

*C*_p _= SSE/*σ*^2 ^+ 2*n*_par _- *n*_obs_≈ SSE/*s*^2 ^_full model _+ 2*n*_par _- *n*_obs _    (9)

(being essentially the same if *σ *is known), and its asymptotic equivalence with leave-one-out (LOO) cross-validation has been demonstrated by Stone [24].

## Results

### Length growth pattern in wild-type fission yeast

**Figure 1:**
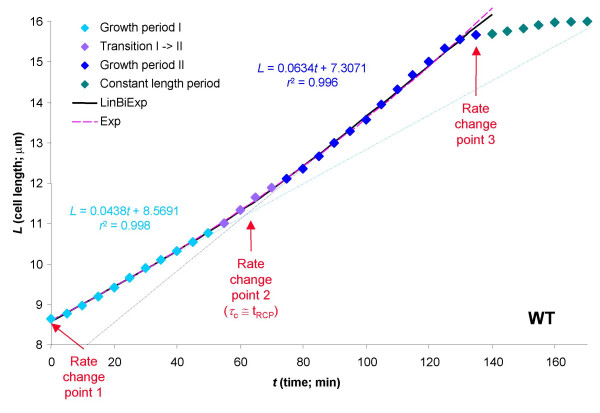
Time-profile of the length-growth in a representative WT fission yeast cell fitted with an exponential (Exp) and a bilinear (LinBiExp) model. Two linear trend-lines fitted separately on the two linear end-segments (denoted by differently colored symbols) are also shown to illustrate the correspondence of the two slopes with those obtained from the bilinear model.

Growth of the wild-type (WT) cell considered is less clearly bilinear as there appears to be no sudden rate-change. Instead, there is a curved middle part corresponding to a transition section (Figure 1). Consequently, the exponential and the bilinear LinBiExp models gave very similar fits that are hard to distinguish visually over most of their ranges. Nevertheless, even on these data, most indicators show LinBiExp, which uses five parameters, to be superior to the more parsimonious exponential model, which uses only two parameters, and they both perform much better than the linear model included here for comparison:

• Linear:    *L = αt + χ *    (10)

*α *= 0.054_(± 0.001)_*μ*m·min^-1^, *χ *= 8.260_(± 0.069)_*μ*m

*n *= 28, *df *= 26, *r*^2 ^= 0.9932, *s *= 0.1886 *μ*m, AIC = 1.81, SBIC = 4.47

• Exponential:    *L *= *αe*^*βt *^    (11)

*α *= 8.605_(± 0.024)_*μ*m, *β *= 0.0046_(± 0.00003) _min^-1^

*n *= 28, *df *= 26, *r*^2 ^= 0.9988, *s *= 0.0761 *μ*m, AIC = -49.03, SBIC = -46.37

• Bilinear (LinBiExp):     

*α*_1 _= 0.042_(± 0.004)_*μ*m·min^-1^, *α*_2 _= 0.064_(± 0.003)_*μ*m·min^-1^,

*χ *= 11.227_(± 0.443)_*μ*m, *τ*_*c *_= 62.62_(± 6.87) _min, *η *= 0.300_(± 0.267)_*μ*m

*n *= 28, *df *= 23, *r*^2 ^= 0.9992, *s *= 0.0680 *μ*m, AIC = -52.74, SBIC = -46.08

*p*_*F *vs. exp _= 0.04

The bilinear model of eq. 12 gives a slightly better performance than the exponential one of eq. 11 as judged from *s *and AIC (they decrease) but not from the more restrictive SBIC, which is more sensitive to the increase in the number of adjustable parameters. According to the *F*-statistics, the improvement in the quality of fit is statistically significant, but just barely below the *p *< 0.05 level [*F*_5–2,23 _= 3.18 (as defined by eq. 4 in the Methods section) ⇒ *p *= 0.04]. The width of the curved transition section of LinBiExp, where it deviates significantly from both its linear segments, is proportional to *η*/(*α*_2 _- *α*_1_); here, data points deviating by more than 0.1 *μ*m from both linear trend-lines were considered as part of the transition section and denoted with a different color (Figure 1). For this particular WT cell, the two slopes obtained from LinBiExp (0.042 *μ*m min^-1^, 0.064 *μ*m min^-1^; eq. 12) correspond to an approximately 50% rate increase and are in excellent agreement with those obtained by separate linear regressions on the two end segments (0.044 *μ*m min^-1^, 0.063 *μ*m min^-1^) as shown in Figure 1. This is somewhat higher than the average of 31% observed for these cells [8], but this is mainly due to the large scattering among individual cells in the population. The position of the RCP at about the 0.36 fraction of the cell cycle (at 62 min with a cycle time of ~ 170 min; eq. 12, Figure 1) is in excellent agreement with the average observed for WT cells (0.34) [8].

### Length growth pattern in *wee1Δ* mutant fission yeast

**Figure 2:**
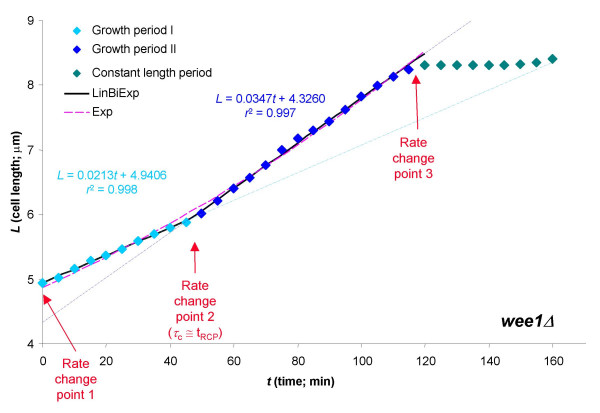
Time-profile of the length-growth in a representative *wee1*Δ fission yeast cell fitted with an exponential (Exp) and a bilinear (LinBiExp) model. As in Figure 1, two linear trend-lines fitted separately on the two linear end-segments (denoted by differently colored symbols) are also shown.

Growth of the representative mutant cell (*wee1*Δ) examined is much more clearly bilinear with a much more abrupt transition (Figure 2); here, consequently, the bilinear model provides a much more clearly superior fit than the exponential model:

• Linear:     *L *= *αt *+ *χ *    (13)

*α *= 0.030_(± 0.001)_*μ*m·min^-1^, *χ *= 4.730_(± 0.047)_*μ*m

*n *= 24, *df *= 22, *r*^2 ^= 0.9880, *s *= 0.1191 *μ*m, AIC = -23.96, SBIC = -21.61

• Exponential:     *L *= *αe*^*βt *^    (14)

*α *= 4.865_(± 0.024)_*μ*m, *β *= 0.0047_(± 0.00006) _min^-1^

*n *= 24, *df *= 22, *r*^2 ^= 0.9960, *s *= 0.0687 *μ*m, AIC = -50.35, SBIC = -47.99

• Bilinear (LinBiExp):     

*α*_1 _= 0.021_(± 0.001)_*μ*m·min^-1^, *α*_2 _= 0.035_(± 0.001)_*μ*m·min^-1^,

*χ *= 5.919_(± 0.074)_*μ*m, *τ*_*c *_= 45.90_(± 2.40) _min, *η *= 0.010_(± 0.075)_*μ*m

*n *= 24, *df *= 19, *r*^2 ^= 0.9992, *s *= 0.0349 *μ*m, AIC = -80.45, SBIC = -74.56

*p*_F vs. exp _= 0.000002

For these data, the difference between the two models and the systematic error of the exponential model are much more pronounced according to all metrics and are much more clearly present even by visual inspection (Figure 2). Consequently, the *F*-statistic also indicates a much more significant difference [*F*_5–2, 19 _= 22.17 ⇒ *p *= 2.0 × 10^-6^] favoring the bilinear profile. Because there seems to be no distinguishable transition section at all, the slopes of the LinBiExp model are in perfect agreement (0.021 *μ*m min^-1^, 0.035 *μ*m min^-1^) with the two individual slopes obtained by linear regression on all points on the left- and right-side of the rate change point (0.021 *μ*m min^-1^, 0.035 *μ*m min^-1^), and they correspond to an approximately 66% rate-increase (somewhat less than the average of 100% observed for these mutants [8]). The rate-change point (*t*_RCP_) is quite clearly delimited and is around 45 min (eq. 15; Figure 2), which corresponds to the 0.28 fraction of the cell cycle, in excellent agreement with the average of 0.27 for these cells. It is also worth noting that the overall growth-rate of the whole cell cycle, (division length – birth length)/cycle time, corresponds to the growth-rate of the first growth period (*α*_1_), as the increased rate in the second growth period after the RCP (*α*_2_) only makes up for the part that is lost during the final, constant-length period. This can clearly be seen in both figures as the first trend-line catches up with the length data exactly at the end of the cycle, so that the rate-growth of the daughter cell(s) will be exactly the same as that of the mother cell, as it should be. For example, in this cell, the overall growth rate is (8.41 *μ*m – 4.94 *μ*m)/160 min = 0.0216 *μ*m min^-1^, which is in good agreement with the corresponding average of (8.4 *μ*m – 5.0 *μ*m)/155 min = 0.0220 *μ*m min^-1 ^obtained from data from 129 cells [8], and corresponds excellently with the growth rate of the first period: *α*_1 _= 0.0213 *μ*m min^-1^.

## Discussion

In balanced growth of asynchronous populations of unicellular organisms, total cell mass increases exponentially as a function of time in parallel with cell number; i.e., both exponential functions are characterized by the same *β *parameter. This also means that every cell (or more precisely, the "average" cell) must double its mass between birth and division. The simplest hypothesis supposes that the size (volume) of individual cells during the cycle grows by the very same exponential function characterized by the very same *β *parameter. The only problem with this hypothesis is that many experiments with different organisms do not support it, and, at least in some cases, linear patterns with one or more rate change point(s) have been found instead [1]. This is a crucial point in cell physiology, since the two pattern-types reflect totally different strategies: namely, exponential growth means that progression through the cell cycle has no effect on growth at all, whereas the existence of rate change point(s) in a linear pattern means that cell cycle (at least at some stages) influences growth at the individual cellular level.

An attractive model organism in these studies is fission yeast, since its length (which is proportional to its volume) can be followed very easily on time-lapse microscopic films. It has long been known that there are at least two rate change points in length growth during the cell cycle of wild-type fission yeast cells [25]. One of them is connected to mitosis; from this point (designated rate change point 3 in Figure 1 and Figure 2) and up to cytokinesis, cell wall synthesis is restricted to septum formation in the middle of the cell leading to a cessation of length growth. After division, the newborn progeny immediately start to grow in length, meaning that there must be another RCP at the beginning of the cycle (designated rate change point 1 in Figure 1 and Figure 2). As a consequence, the cell cycle definitely influences length growth in fission yeast; however, whether or not growth is exponential between RCP1 and RCP3 remains an open question. Experiments seem to favor a bilinear pattern with a third RCP (designated as rate change point 2 in Figure 1 and Figure 2) over an exponential one [6,8,9]; however, detailed statistical analysis has been lacking.

**Figure 3:**
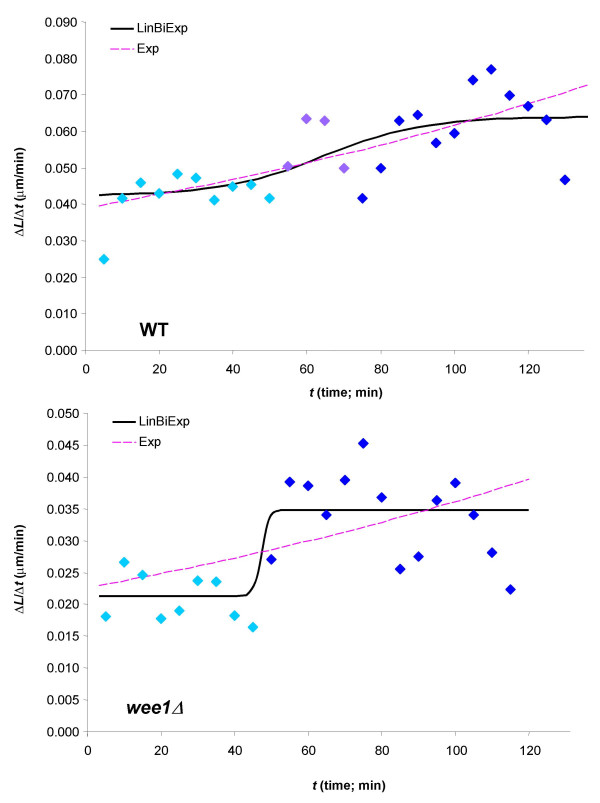
Time-profiles of the speed (rate) of length-growth (Δ*L*/Δt for the experimental data and d*L*/d*t*, the first order derivative, for the model functions) for the two types of cells investigated here, together with those obtained from the best-fitting exponential (Exp) and bilinear (LinBiExp) models.

Because there is only a relatively limited range for both the dependent (*L*) and the independent (*t*) variables in the cases considered here, the statistical evidence suggesting a bilinear dependence rather than an exponential one is not strong enough to favor one model unequivocally over the other. Nevertheless, the bilinear time-profile seems more adequate according to model selection criteria standards, as described in the Methods section, especially in the case of the *wee1*Δ cells. This is also well illustrated by a comparison of the predicted speeds of length-growth in the best-fitting exponential and bilinear models (Figure 3): the characteristic sigmoid step-up profile obtained from the bilinear model fits the experimental data for *wee1*Δ much better than the continuously increasing profile obtained from the exponential model, but the case of the WT is less clear.

A major goal of the present paper is to propose a general quantitative framework for judging the adequacy of bilinear versus exponential models for arbitrary growth profiles. Hopefully, in addition to the relatively limited number of applications included here, the present detailed description of quantitative model selection procedures will also help to differentiate accurately among linear, exponential and bilinear models for future cell growth data. Furthermore, by introducing the fully optimizable bilinear model LinBiExp, the cumbersome approach of performing two separate linear regressions after separating the data at a visually determined place can be replaced by a single, unified fitting. Hence, the nonlinear regression algorithm itself will determine the position of the rate change point (*t*_RCP_) and the value of the two slopes on its left and right sides (*α*_1_, *α*_2_, respectively) by minimizing the sum of squared errors (SSE), and this will not have to be done by the user on the basis of preconceived assumptions or mere visual inspection. To facilitate the application of these models and model selection criteria further, a fully functional Excel worksheet-based implementation, which relies on Excel's powerful Solver data analysis tool and contains detailed instructions, is included as a downloadable supplement (see additional file 1: Excel spreadsheet with the *wee1*Δ data used to perform this analysis.)

Finally, we are certain that from a cell biologist's perspective, it might be difficult to accept that a mutant shows a particular phenomenon more clearly than the wild type. In such cases, the effect of the mutation on the observed phenomenon should also be examined. We are fortunate to be able to say that the bilinear length growth pattern of fission yeast is probably not an artifact produced somehow by deleting the *wee1 *gene from the genome. Formerly, we assumed that the reason for the existence of RCP2 in WT is different from that in the *wee1*Δ mutant [10]. At about 1/3^rd ^of their cycle, WT cells are in mid-G2 phase; they are just passing through the so-called mitotic checkpoint and are changing from unipolar to bipolar growth (a phenomenon called new end take-off, NETO, see [6]). It is easy to imagine that the RCP caused by NETO is not a sharp one, since the growth rate at the new end may continuously increase for a period. In contrast, the small-sized *wee1*Δ mutant cells have a quite different type of cell cycle: at about 1/4^th ^of their cycle, they are just replicating their DNA [26], which is a fast process on the scale of the whole cycle. As a consequence, S phase could cause the rate change here via the gene dosage effect, which might be a much sharper process, leading to a clear bilinear pattern. Note that the rate increase at RCP2 is also larger in the *wee1*Δ mutant than in wild type [8].

## Competing interests

The author(s) declare that they have no competing interests.

## Authors' contributions

PB conceived the study, carried out the calculations, and drafted the manuscript. AS carried out the original cell length measurements, helped in the interpretation of the model results, and completed the manuscript. Both authors read and approved the final manuscript.

## Supplementary Material

Additional File 1 Click here for file
